# Flow inefficiencies in non-obstructive HCM revealed by kinetic energy and hemodynamic forces on 4D-flow CMR

**DOI:** 10.1093/ehjimp/qyae074

**Published:** 2024-07-16

**Authors:** K Pola, Z Ashkir, S Myerson, H Arheden, H Watkins, S Neubauer, P M Arvidsson, B Raman

**Affiliations:** University of Oxford Centre for Clinical Magnetic Resonance Research, Division of Cardiovascular Medicine, Radcliffe Department of Medicine, University of Oxford, Oxford, United Kingdom; Lund University, Skåne University Hospital Lund, Department of Clinical Sciences Lund, Clinical Physiology, Lund, Sweden; University of Oxford Centre for Clinical Magnetic Resonance Research, Division of Cardiovascular Medicine, Radcliffe Department of Medicine, University of Oxford, Oxford, United Kingdom; University of Oxford Centre for Clinical Magnetic Resonance Research, Division of Cardiovascular Medicine, Radcliffe Department of Medicine, University of Oxford, Oxford, United Kingdom; Lund University, Skåne University Hospital Lund, Department of Clinical Sciences Lund, Clinical Physiology, Lund, Sweden; University of Oxford Centre for Clinical Magnetic Resonance Research, Division of Cardiovascular Medicine, Radcliffe Department of Medicine, University of Oxford, Oxford, United Kingdom; University of Oxford Centre for Clinical Magnetic Resonance Research, Division of Cardiovascular Medicine, Radcliffe Department of Medicine, University of Oxford, Oxford, United Kingdom; University of Oxford Centre for Clinical Magnetic Resonance Research, Division of Cardiovascular Medicine, Radcliffe Department of Medicine, University of Oxford, Oxford, United Kingdom; Lund University, Skåne University Hospital Lund, Department of Clinical Sciences Lund, Clinical Physiology, Lund, Sweden; University of Oxford Centre for Clinical Magnetic Resonance Research, Division of Cardiovascular Medicine, Radcliffe Department of Medicine, University of Oxford, Oxford, United Kingdom

**Keywords:** non-obstructive hypertrophic cardiomyopathy, cardiovascular magnetic resonance, 4D flow, kinetic energy, hemodynamic forces

## Abstract

**Aims:**

Patients with non-obstructive hypertrophic cardiomyopathy (HCM) exhibit myocardial changes which may cause flow inefficiencies not detectable on echocardiogram. We investigated whether left ventricular (LV) kinetic energy (KE) and hemodynamic forces (HDF) on 4D-flow cardiovascular magnetic resonance (CMR) can provide more sensitive measures of flow in non-obstructive HCM.

**Methods and results:**

Ninety participants (70 with non-obstructive HCM and 20 healthy controls) underwent 4D-flow CMR. Patients were categorized as phenotype positive (P+) based on maximum wall thickness (MWT) ≥ 15 mm or ≥13 mm for familial HCM, or pre-hypertrophic sarcomeric variant carriers (P−). LV KE and HDF were computed from 4D-flow CMR. Stroke work was computed using a previously validated non-invasive method. P+ and P− patients and controls had comparable diastolic velocities and LV outflow gradients on echocardiography, LV ejection fraction, and stroke volume on CMR. P+ patients had greater stroke work than P− patients, higher systolic KE compared with controls (5.8 vs. 4.1 mJ, *P* = 0.0009), and higher late diastolic KE relative to P− patients and controls (2.6 vs. 1.4 vs. 1.9 mJ, *P* < 0.0001, respectively). MWT was associated with systolic KE (*r* = 0.5, *P* < 0.0001) and diastolic KE (*r* = 0.4, *P* = 0.005), which also correlated with stroke work. Systolic HDF ratio was increased in P+ patients compared with controls (1.0 vs. 0.8, *P* = 0.03) and correlated with MWT (*r* = 0.3, *P* = 0.004). Diastolic HDF was similar between groups. Sarcomeric variant status was not associated with KE or HDF.

**Conclusion:**

Despite normal flow velocities on echocardiography, patients with non-obstructive HCM exhibited greater stroke work, systolic KE and HDF ratio, and late diastolic KE relative to controls. 4D-flow CMR provides more sensitive measures of haemodynamic inefficiencies in HCM, holding promise for clinical trials of novel therapies and clinical surveillance of non-obstructive HCM.

## Graphical Abstract

**Graphical Abstract:**
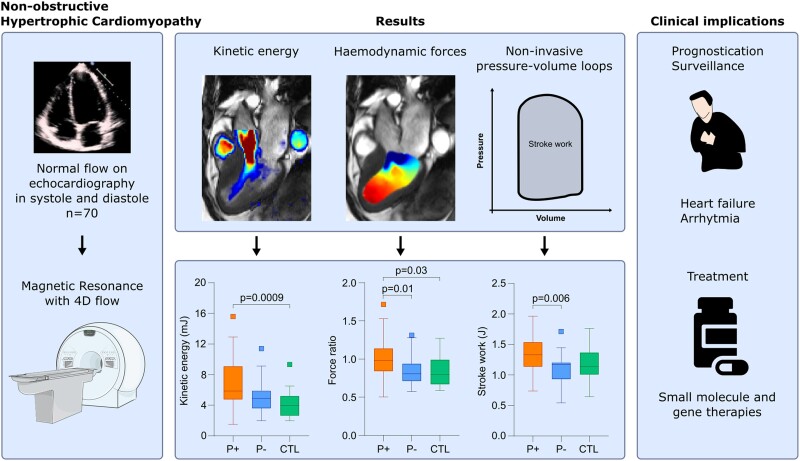

## Introduction

Hypertrophic cardiomyopathy (HCM) is a complex genetic disease associated with an increased lifetime burden of symptoms.^1^ Although left ventricular outflow tract (LVOT) obstruction is a frequent manifestation, ∼30–40% have a non-obstructive phenotype.^2^ Echocardiography is the workhorse for the assessment of patients with HCM, specifically for exclusion of significant outflow gradients or evaluation of diastolic impairment.^3^ However, Doppler echocardiography has been shown to have a poor to modest correlation with invasive measures of LV filling pressures, in part due to the dependency on patient and operator factors.^4^

Four-dimensional flow cardiovascular magnetic resonance (4D-flow CMR) has the potential to overcome many of these limitations, as it permits three-dimensional assessment of ventricular and atrial blood flow throughout the cardiac cycle. Several studies, albeit small, have examined the role of 4D-flow CMR to characterize flow perturbations in numerous cardiac diseases, including obstructive HCM.^5^ Abnormalities in ventricular flow have been linked to an increased risk of heart-failure symptoms and embolic complications.^6,7^ In contrast, the role of such measures in non-obstructive HCM, where echocardiography may come with limitations, is less well studied. We recently demonstrated the utility of flow-component analysis on 4D-flow CMR in patients with non-obstructive HCM.^8^ Our findings suggest that such patients had marked inefficiencies in intraventricular flow, which warrant further investigation.

In recent times, there have been numerous efforts to assess the three-dimensional forces exchanged between the blood pool and the surrounding myocardium, also known as hemodynamic forces (HDF). Hemodynamic-force analysis conveys valuable information about the coupling of ventricular motion and the resulting blood-flow patterns.^9–11^ In a healthy person, LV HDF during systole are typically larger in magnitude in the longitudinal (apex to base) direction than in the transverse plane (lateral wall to septum and anterior to inferior directions). Conversely, the failing heart may exhibit a higher transverse to longitudinal force ratio, which has been suggested as a clinical marker of inefficient ventricular flow.^12^

Similarly, one can also comprehensively evaluate the energy of the intraventricular blood due to its motion, also known as kinetic energy (KE), which is calculated from the mass and velocity of the blood within the ventricle. Notable variations in systolic and diastolic ventricular KE have been described in health and disease. The healthy left ventricle produces peak systolic KE of up to 3.1–4.9 mJ,^13–15^ while diastolic KE exhibits age-dependence and is closely correlated with diastolic function.^15–17^ Previous studies have shown that patients with dilated cardiomyopathy and reduced ejection fraction tend to have normal or high systolic KE, indicating an increased ventricular workload,^15,18,19^ whereas individuals with heart failure and preserved ejection fraction typically exhibit an increased diastolic KE relative to controls.^15^ The efficiency of the ventricle can be illustrated by its capability to transform metabolic energy into mechanical work, which in turn is converted to the KE of propelling blood. This process culminates in the effective ejection of blood. By evaluating both LV stroke work and KE, we gain insights into the mechanical efficiency of the heart.

There is currently an unmet need for more sensitive measures of flow perturbations in non-obstructive HCM and pre-hypertrophic sarcomeric variant carriers, given the limited sensitivity of echocardiography. The aim of this study was to evaluate the utility of HDF and KE, two sensitive measures from 4D-flow CMR that carry complementary information about systolic and diastolic myocardial-blood flow coupling flow, in detecting flow abnormalities in the early stages of HCM without left ventricular outflow tract obstruction.

## Methods

**Figure 1:**
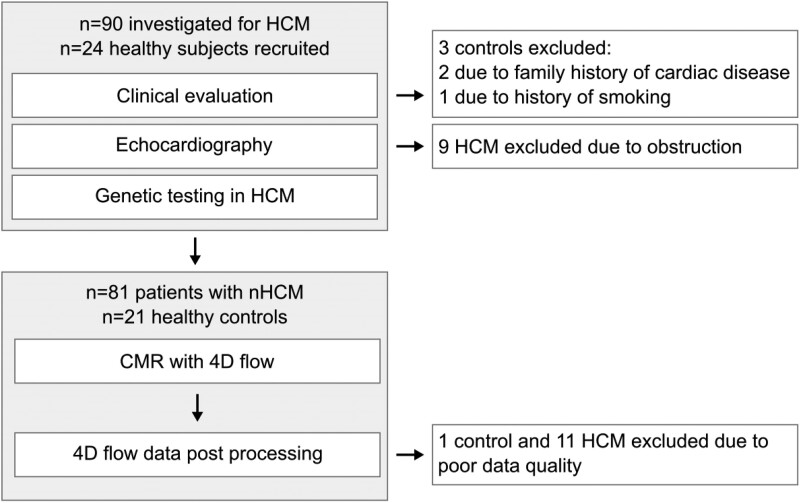
Overview of the study design. CMR, cardiac magnetic resonance; nHCM, non-obstructive hypertrophic cardiomyopathy.

This prospective observational study included patients with HCM investigated at our tertiary centre between 2016 and 2018. The patients were invited as part of a research study, and an overview of the study design is shown in *Figure 1*.

Patient inclusion criteria were: non-obstructive HCM or pre-hypertrophic sarcomeric variant carriers. All patients had to have a resting and post-Valsalva LV outflow tract gradient < 30 mmHg to be eligible.

Exclusion criteria were: contra-indications to CMR, presence of LVOT obstruction, HCM phenocopies (e.g. hypertensive heart disease, mitochondrial diseases, and Fabry disease), moderate/severe valve disease, significant concomitant coronary artery disease, and persistent atrial fibrillation.

The patients were categorized as phenotype positive (P+) based on the presence of hypertrophy on CMR (maximum wall thickness ≥ 15 mm, or ≥13 mm with a positive family history), or as phenotype negative (P−) referring to pre-hypertrophic pathogenic or likely pathogenic sarcomeric variant carriers, as previously described.^8,21^ Based on the pattern of hypertrophy, the patients were further categorized by two expert readers (>5 years of clinical experience in inherited cardiac conditions) in line with prior recommendations,^20^ into hypertrophy phenotype subgroups: septal, reverse septal, apical, or concentric.

Healthy volunteers were prospectively enrolled as a control group and matched for age and sex to the patient cohort at the group level. Exclusion criteria for the controls were known cardiovascular disease, use of cardiovascular medications, or a family history of significant cardiac disease.

### Echocardiography

All patients with non-obstructive HCM underwent a clinical echocardiogram prior to study enrolment to meet study eligibility. On the day of examination, research echocardiograms were performed in all participants after the CMR scan to assess the pressure gradients in the LVOT and the LV diastolic function using two-dimensional transthoracic echocardiography (EPIQ7, Philips, Netherlands). Pulsed wave Doppler was used for *trans*-mitral measurements of early (E) and late (A) diastolic filling velocities, e-wave deceleration time, and E/A ratio. Tissue Doppler imaging was used to measure early diastolic lateral and septal mitral annular velocities (e′).

### CMR scan

A 3T (Trio, Siemens) scanner was used to acquire cardiac magnetic resonance imaging and flow data. Assessments of cardiac volumes and function were acquired using standard retrospective ECG-gated short- and long-axis balanced steady-state free precession (bSSFP) cine images. Typical acquisition parameters for the cine bSSFP images were as follows: echo time 1.12 ms, temporal resolution 35–40 ms, acquired spatial resolution 1.25 mm^2^, flip angle 50°, and slice thickness 8 mm. This was followed by acquisition of time-resolved three-dimensional phase-contrast flow (4D flow) images from a box covering the heart and proximal great vessels, using a gradient-echo sequence with Cartesian readout and free breathing with respiratory gating.^21^ The total time of acquisition ranged from 10 to 20 min depending on the heart rate and breathing pattern. Typical 4D-flow sequence parameters were: echo time 2.75 ms, repetition time 4.3 ms, temporal resolution 52 ms, acquired spatial resolution 3 mm isotropic, flip angle 7°, and acceleration method SENSE factor 2. Cine bSSFP images and 4D-flow images were acquired pre-contrast, followed by late gadolinium enhancement (LGE) imaging in patients for the assessment of myocardial-fibrosis burden as previously described.^22^

### Post-processing of 4D-flow data

Phase background correction was performed using fitting to stationary tissue,^23^ and aliasing errors were corrected by automatic phase unwrapping.^24^ The spatial orientation of the cine images was manually adjusted to align with the 4D-flow data. To facilitate visual comparison of KE and HDF between hearts with varying heart rates, a common time axis was created by linear resampling using end diastole and end systole as temporal landmarks.

### CMR data analysis

All image analyses were performed using the software Segment v3.3 R10057 (Medviso, Lund, Sweden).^25^ Left ventricular volumes were defined by contouring of the endocardium in the short-axis cine bSSFP images for the entire cardiac cycle, including papillary muscles and trabeculations to the blood-pool volume according to recommendations.^26^ The time-resolved contouring of the LV volumes was interpolated from the short-axis bSSFP images to the 4D-flow data to define the volume of interest for the 4D-flow analyses. End diastole and end systole were defined by planimetry as the largest and smallest volumes, respectively, in the short-axis images. Left ventricular mass was measured by manual contouring in both end diastole and end systole and calculated by multiplying the myocardial volume with a density of 1.05 g/cm^3^. The extent of focal fibrosis was evaluated from LGE images using the thresholding method where pixels with a signal intensity > 5 SD from a normal reference region of interest enabled LGE mass estimation.

### Kinetic energy

Kinetic energy was calculated for each voxel in the LV throughout the cardiac cycle as previously described,^13,14^ using the following formula:


$$\mathrm{KE}=\frac{\mathrm{mass}\;\times \;\mathrm{velocit}y^{2}}{2}.$$

The instantaneous magnitude of the velocity vector (speed) for each voxel was computed from the 4D-flow images. Total KE in each time frame was then calculated as the sum of KE in all voxels within the LV cavity. Measurements of KE were analysed using peak values for systole and early and late diastole.

### Hemodynamic forces

Hemodynamic forces were analysed using a validated method previously described in detail.^10,27^ In summary, instantaneous pressure gradients were calculated from CMR 4D-flow data using the Navier–Stokes equations and integrated over the LV cavity for each point in time. Intraventricular HDF were analysed as three perpendicular components, defined using a spatial reference system based on the position of the atrioventricular (AV) plane at end diastole. Measurements of HDF were calculated for systole and diastole as root mean square values, to facilitate comparison of force magnitudes regardless of vector direction. Hemodynamic force ratio was used as a quantitative measurement of the principal direction of HDF, and calculated as the sum of the two transverse components divided by the longitudinal component, using the following formula:


$$\mathrm{HDF}\;\mathrm{ratio}=\frac{\sqrt{\mathrm{lateral}\;\mathrm{wall}\text{–}\mathrm{septu}m^{2}+\mathrm{inferior}\text{–}\mathrm{anterio}r^{2}}}{\mathrm{apex}\text{–}\mathrm{base}}.$$

### Validation of analysis methods for KE and HDF

A method agreement study of KE and HDF was performed using two different methods for contouring of the LV cavity. Some patients with HCM have extensive papillary muscles and trabeculations, and these were included in the LV cavity in the standard contouring according to recommendations.^26^ Papillary-border time-resolved contouring excluded papillary muscles and trabeculations from the LV cavity. Validation was performed in seven study participants (five patients and two controls) to investigate the impact of contouring with and without papillary muscles and trabeculations included in the LV cavity on measurements of KE and HDF.

### Estimation of stroke work from validated non-invasive pressure–volume loops

Pressure–volume loops were generated from CMR and a concurrent brachial blood pressure measurement using a previously validated method.^28^ Briefly, the time-resolved LV volume curve was combined with a time-varying elastance function to compute an LV pressure curve. The elastance function was scaled in amplitude to the brachial blood pressure and in time so that the steepest point of pressure decline coincides with end systole. Estimated end-diastolic pressure was set to 7.5 mmHg as previously suggested. Stroke work was measured as the area of the resulting pressure–volume loop.

### Statistical analysis

Statistical analysis was performed using Prism v. 9.4.1 (GraphPad Software, La Jolla, CA, USA). Continuous data are presented as mean and standard deviation or median and interquartile range, and categorical data as absolute numbers and proportion (%). The Student’s *t*-test and the Mann–Whitney *U* test were used for group comparisons where relevant, Spearman analysis for correlations, Fisher’s exact test for binary categorical data, and Bland–Altman for method agreement. A two-tailed *P* < 0.05 was considered significant. When multiple tests were performed, statistical significance was ascertained after correcting for multiple testing by dividing the significance level of the *P*-value by the number of tests. Multivariate linear and logistic regression analyses were undertaken using SPSS version 29.0 to examine if associations between phenotypic traits, genotype, and 4D-flow parameters were independent of age, sex, and BMI variations.

## Results

### Patient characteristics

In total, 81 patients and 21 controls met the inclusion criteria, and 11 (14%) patients and 1 (5%) control were excluded due to inadequate 4D-flow data quality (*Figure 1*). The final study cohort comprised of 90 participants, which included 70 patients, of which 48 (69%) were HCM phenotype positive (P+) and 22 (31%) pre-hypertrophic variant carriers (P−), and 20 controls (*Table 1*). When stratifying the P+ patients according to hypertrophy phenotype, 14 (29%) had septal hypertrophy, 14 (29%) reverse septal, 9 (19%) apical, and 11 (23%) concentric. Thirty-two (65%) of the P+ patients harboured a sarcomeric variant. There was no patient with apical aneurysm. Age and sex were similar between the P+ patients and the controls, but the P− patients were younger and had greater female representation at the group level. The P+ patients had greater BMI compared with the P− patients and the controls.

**Table 1:** Characteristics in P+ and pre-hypertrophic P− patients with HCM, and controls

	P+	P−	Controls	*P* value P+ vs. P−	*P* value P+ vs. controls	*P* value P− vs. controls
Characteristics						
*N*	48	22	20			
Women; *n*, (%)	8 (16)	11 (50)	7 (35)	*0*.*008*	0.1	0.4
Age, years^#^	51 [13]	35 [14]	47 [20]	*<0*.*0001*	0.3	*0*.*03*
BMI, kg/m^2#^	27 [4.5]	24 [4.8]	24 [2.2]	*0*.*003*	*0*.*0008*	0.9
Systolic blood pressure, mmHg^#^	126 [14]	114 [14]	121 [14]	*0*.*001*	0.2	0.1
Diastolic blood pressure, mmHg^#^	75 [12]	69 [11]	70 [10]	0.08	0.1	0.8
ESC risk score^#^	2.5 [0.4]	1.9 [0.4]		*<0*.*0001*		
Sarcomeric mutation, *n* (%)	32 (65)	22 (100)				
CMR findings						
Max wall thickness, mm^#^	21 [5]	12 [2]	11 [2]			*0*.*004*
Heart rate, bpm^#^	61 [7]	62 [11]	67 [11]	0.8	*0*.*02*	0.1
Stroke volume, mL^#^	107 [20]	100 [24]	99 [25]	0.2	0.1	0.9
Stroke volume/BSA, mL/m^2#^	54 [9]	55 [11]	55 [13]	0.5	0.5	1.0
LVEF, %^#^	60 [7]	60 [5]	60 [5]	0.9	0.9	0.9
Stroke work (J)^#^	1.3 [0.3]	1.1 [0.3]	1.2 [0.4]	*0*.*002*	0.051	0.6
LV mass, g^#^	138 [38]	91 [22]	90 [27]	*<0*.*0001*	*<0*.*0001*	0.9
LV mass/BSA, g/m^2#^	69 [20]	50 [11]	51 [13]	*0*.*0001*	*0*.*0006*	1.0
End-diastolic volume, mL^#^	181 [32]	168 [45]	166 [43]	0.2	0.1	0.9
End-diastolic volume/BSA, mL/m^2#^	90 [15]	93 [20]	93 [22]	0.5	0.5	1.0
End-systolic volume, mL^#^	74 [21]	68 [23]	67 [21]	0.3	0.2	0.9
End-systolic volume/BSA, mL/m^2#^	37 [11]	38 [11]	38 [11]	0.7	0.7	1.0
Cardiac output, L/min^#^	6.5 [1.2]	6.0 [1.0]	6.5 [1.4]	0.07	0.9	0.2
LGE total mass, g	5.0	0.3		*<0*.*0001*		
LGE relative enhanced volume, %	0.047 [0.019–0.10]	0.0044 [0.0019–0.011]		*<0*.*0001*		
Echocardiographic findings						
E, cm/s^#^	68 [19]	74 [20]	70 [13]	0.2	0.7	0.5
A, cm/s^#^	58 [17]	60 [15]	59 [12]	0.9	0.8	0.7
S/D ratio^#^	1.2 [0.42]	1.2 [0.42]	1.2 [0.31]	0.7	0.8	0.6
DTs^#^	0.20 [0.052]	0.19 [0.057]	0.19 [0.027]	0.5	0.4	0.9
Average e′, cm/s^#^	8.0 [3.2]	10 [4.1]	11 [3.5]	*0*.*04*	*0*.*02*	0.7
Average E/e′ ratio^#^	9.0 [2.9]	8.3 [3.5]	7.0 [1.6]	0.4	*0*.*02*	0.2
E/A ratio^#^	1.2 [0.26]	1.4 [0.58]	1.2 [0.26]	0.3	0.8	0.3
LVOT max gradient, mmHg^#^	6.0 [3.4]	6.0 [2.8]	5.2 [3.3]	1.0	0.5	0.5
Medication						
ACEi/ARB; *n*, (%)	5 (10)	0	0	0.3		
Beta blocker; *n*, (%)	20 (41)	4 (18)	0	0.1		
Calcium channel blocker; *n*, (%)	10 (20)	0	0	*0*.*03*		

Data are expressed as median and [interquartile range], mean and [standard deviation]^#^, or absolute numbers and (%). *P*-values <0.05 are noted in italics.

BMI, body mass index; BSA, body surface area; DT, deceleration time; D, diastolic velocity; E, early diastolic mitral valve inflow velocity; e′, early diastolic tissue velocity; LV, left ventricle; LGE, late gadolinium enhancement; LVOT, left ventricular outflow tract; S, systolic velocity.

### Echocardiography

Maximal LVOT pressure gradients on echocardiography were minimal and comparable between the P+ patients, the P− patients and the controls (*Table 1*). There were no differences in E, A, systolic to diastolic pulmonary vein Doppler (S/D) ratio, DTs or E/A ratio between the three groups. The P+ patients had lower average e′ on tissue Doppler, and thus higher average E/e′ ratio relative to the controls. However, all patients had E/e′ ratios within normal limits (<14).

### CMR

There were no differences in LV stroke volume, ejection fraction, end-diastolic and end-systolic volume, or cardiac output between the P+ and P− patients and the controls (*Table 1*). Maximum wall thickness was 21 ± 5 mm in P+, 12 ± 2 mm in P−, and 11 ± 2 mm in controls. As expected, the P+ patients had greater LV mass compared with the P− patients and the healthy controls. LGE mass was greater in the P+ patients compared with the P− patients. The P+ patients had higher European Society of Cardiology (ESC) estimated risk score for sudden cardiac death (SCD) compared with the P− individuals (*Table 1*), although on average this was deemed to be low.

### Stroke work

Stroke work was significantly higher in the P+ patients compared with the P− patients and the controls, despite similar stroke volume across groups. In particular, patients with septal hypertrophy had the highest stroke work of all subgroups (*Table 1* and Supplementary data online, *Table S1*). In the group of all patients with HCM, stroke work correlated with KE in systole (*r* = 0.6 *P* < 0.0001), early diastole (*r* = 0.3 *P* = 0.008) and late diastole (*r* = 0.4 *P* = 0.003), as well as with systolic HDF in the apex–base (*r* = 0.5 *P* < 0.0001), lateral wall–septum (*r* = 0.5 *P* < 0.0001) and inferior–anterior (*r* = 0.4 *P* = 0.002) directions, and with diastolic HDF in the apex–base (*r* = 0.5 *P* = 0.0002) and inferior–anterior (*r* = 0.5 *P* = 0.0002) directions, but not with HDF transverse/longitudinal ratio in systole or diastole (*P* > 0.8). After correcting for multiple testing (i.e. *P* < 0.005), the correlation between stroke work and systolic and late diastolic KE and systolic and diastolic HDF (in all directions) remained statistically significant.

### Kinetic energy and haemodynamic forces

**Figure 2:**
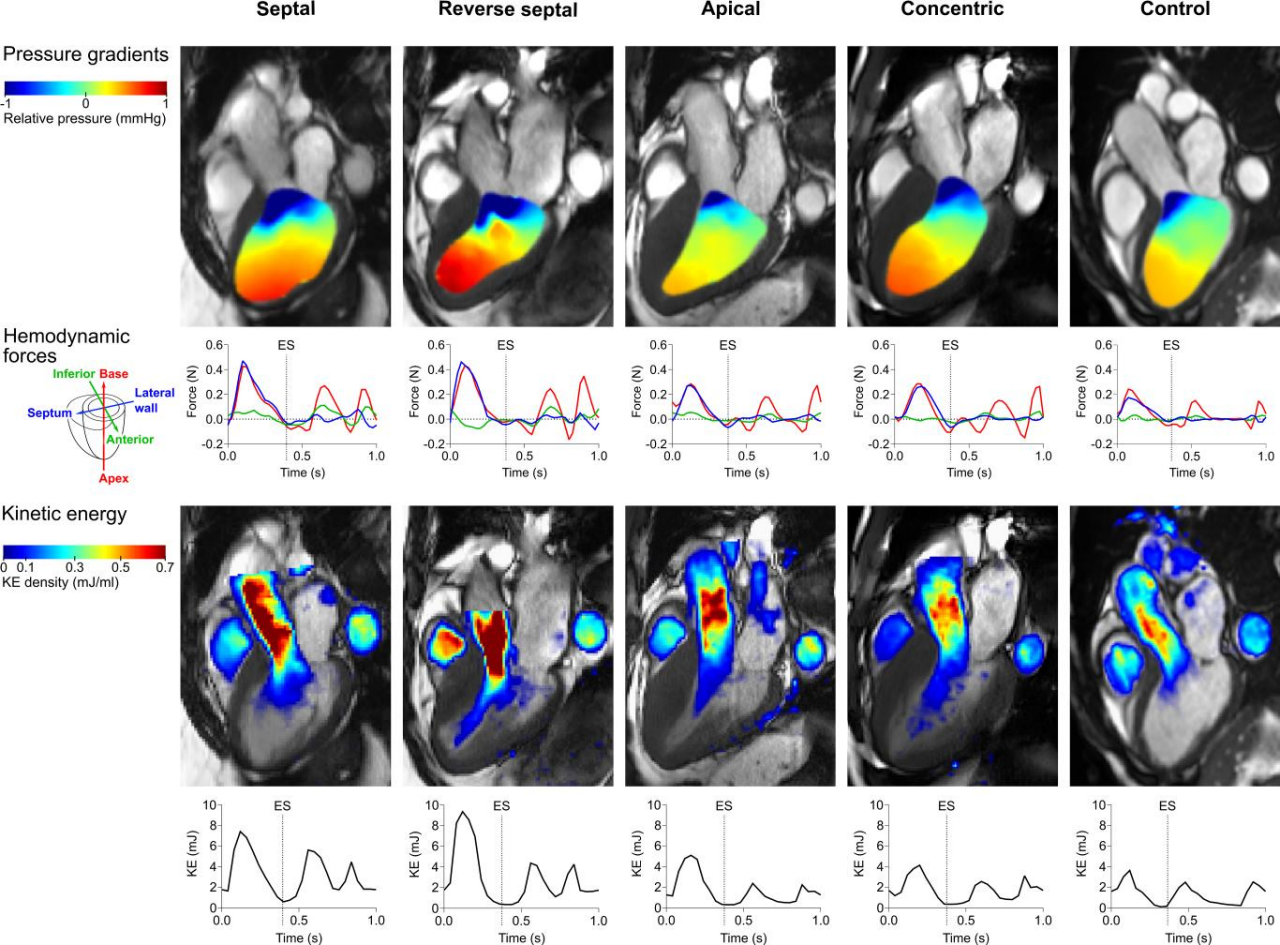
Visualization of left ventricular pressure gradients, HDF, and KE in HCM phenotype subgroups: septal, reverse septal, apical, and concentric, and in the controls. ES, end systole. End diastole is timepoint 0.

**Figure 3:**
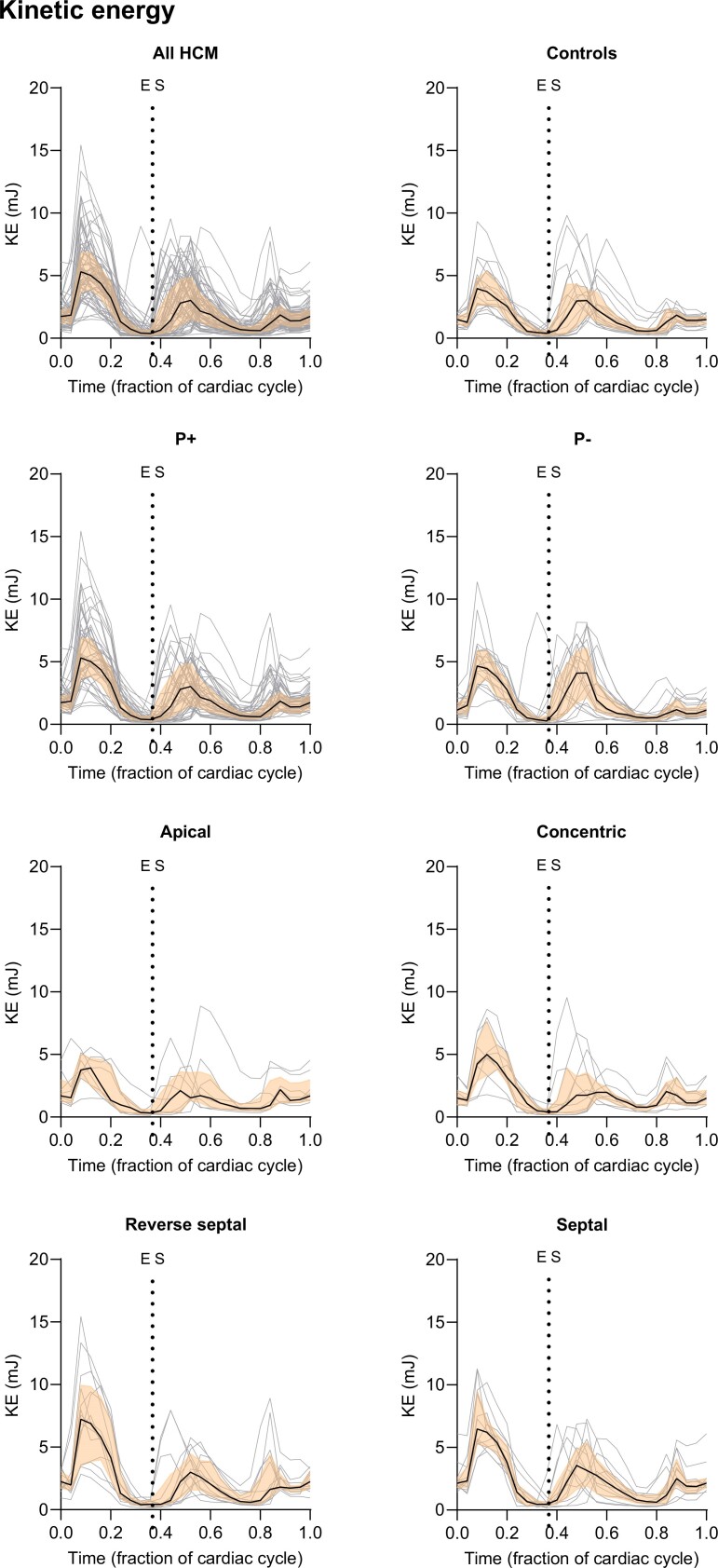
Kinetic energy over the cardiac cycle in hypertrophy phenotype subgroups. Data are presented as individual curves in shaded grey, and median (black) and interquartile range (transparent orange area) for each time phase. HCM, hypertrophic cardiomyopathy; P+, phenotype positive; P−, phenotype negative; ES, end systole. End diastole is timepoint 0.

**Figure 4:**
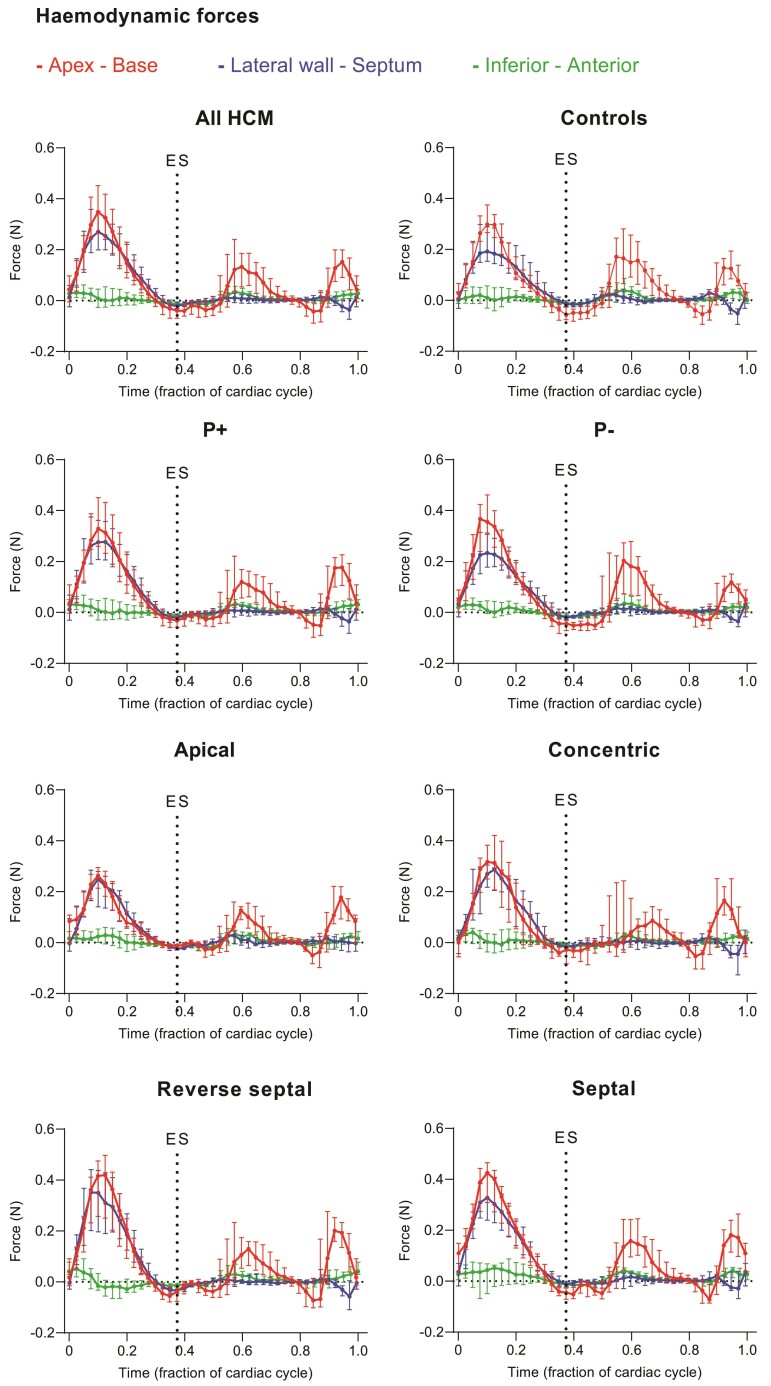
Hemodynamic forces over the cardiac cycle in hypertrophy phenotype subgroups. Data are expressed as median and interquartile range for each time phase. HCM, hypertrophic cardiomyopathy; P+, phenotype positive; P−, phenotype negative; ES, end systole. End diastole is timepoint 0.

*Figure 2* shows a visualization of LV pressure gradients, HDF, and KE in each of the studied groups. Method agreement between two different contouring strategies for KE and HDF measures was excellent (see Supplementary data online, *Figure S1*). Left ventricular KE traces over the entire cardiac cycle for all patients and controls are presented in *Figure 3*. Hemodynamic force traces in three perpendicular directions over the entire cardiac cycle are presented in *Figure 4*.

**Figure 5:**
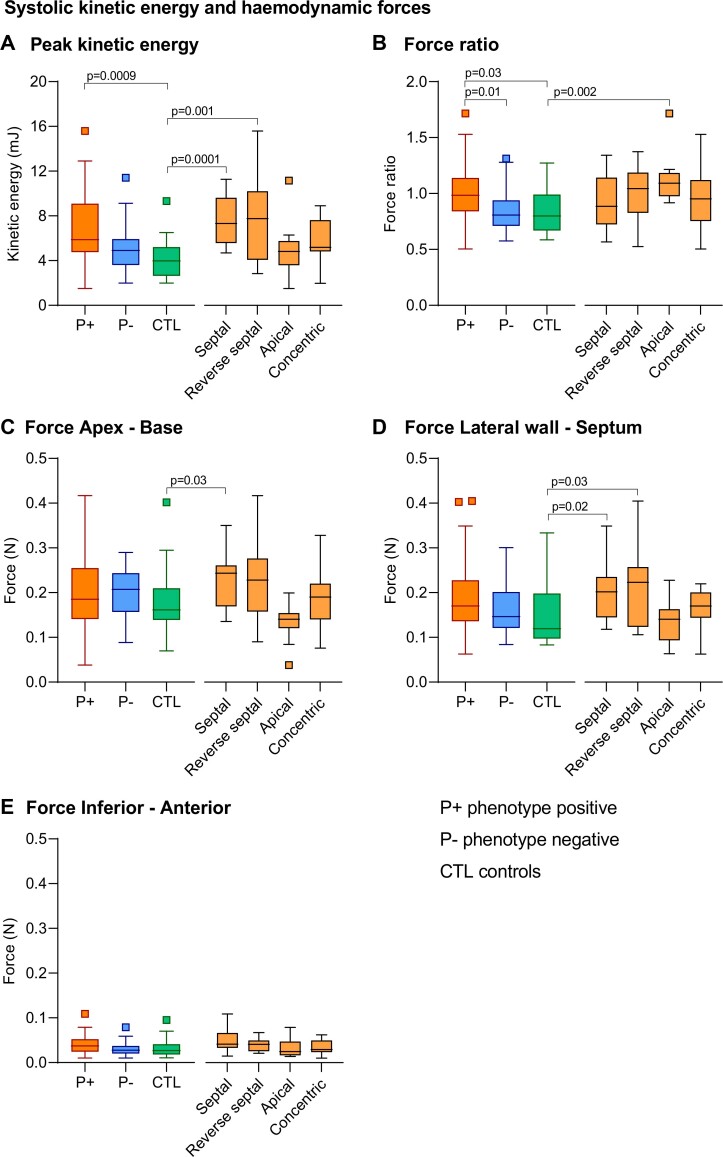
Systolic peak kinetic energy (*A*), HDF ratio (*B*) and root mean square HDF (*C-E*) in patients with non-obstructive P+ HCM, P− HCM, and controls (CTL), and according to hypertrophy phenotype subgroups.

Peak KE and root mean square HDF and HDF ratio for systole and diastole are presented in *Table 2* and Supplementary data online, *Table S1* and *Figure 5*.

**Table 2:** Hemodynamic forces and peak KE in P+ and pre-hypertrophic P− patients with HCM, and controls

	P+	P−	Controls	*P* value P+ vs. P−	*P* value P+ vs. controls	*P* value P− vs. controls
Peak kinetic energy (mJ)						
Systole	5.8 [4.6–8.9]	5.1 [3.8–6.0]	4.1 [2.7–5.4]	0.06	*0*.*0009*	0.1
Early diastole	4.1 [2.8–5.7]	6.3 [4.4–7.6]	4.1 [2.9–5.8]	*0*.*006*	0.7	0.08
Late diastole	2.6 [1.9–4.0]	1.4 [1.1–2.1]	1.9 [1.4–2.3]	*<0*.*0001*	*0*.*002*	0.09
Peak kinetic energy indexed to end-diastolic volume (mJ/L)						
Systole	32 [27–45]	32 [25–39]	23 [19–32]	0.2	*0*.*0009*	0.07
Early diastole	25 [17–32]	34 [27–47]	28 [18–36]	*0*.*0005*	0.2	0.051
Late diastole	16 [10–22]	8.7 [7.0–12]	11 [7.9–16]	*0*.*0003*	*0*.*02*	0.1
Hemodynamic forces^a^ (N)						
Longitudinal (apex–base)						
Systole	0.18 [0.14–0.25]	0.20 [0.16–0.24]	0.16 [0.14–0.22]	0.7	0.3	0.2
Diastole	0.11 [0.087–0.14]	0.12 [0.097–0.15]	0.12 [0.084–0.14]	0.4	0.6	0.8
Transverse (lateral wall–septum)						
Systole	0.17 [0.14–0.23]	0.15 [0.12–0.20]	0.12 [0.11–0.20]	0.2	0.051	0.3
Diastole	0.027 [0.020–0.035]	0.025 [0.017–0.029]	0.030 [0.023–0.035]	0.1	0.5	*0*.*04*
Transverse (inferior–anterior)						
Systole	0.034 [0.023–0.050]	0.027 [0.020–0.032]	0.025 [0.018–0.039]	0.07	0.1	0.9
Diastole	0.026 [0.022–0.033]	0.029 [0.021–0.034]	0.022 [0.018–0.034]	0.9	0.4	0.6
Hemodynamic force ratio (transverse/longitudinal)						
Systole	1.0 [0.83–1.1]	0.82 [0.71–0.95]	0.83 [0.72–0.96]	*0*.*01*	*0*.*03*	0.7
Diastole	0.36 [0.27–0.45]	0.30 [0.27–0.36]	0.34 [0.30–0.45]	0.09	0.6	*0*.*03*

Data are expressed as median and [interquartile range]. *P*-values <0.05 are noted in italics.

^a^Mean and peak early and late diastolic HDF could not be computed reliably, and therefore root mean square pan-diastolic HDF and HDF ratio were used.

### Systolic and diastolic kinetic energy

Peak systolic KE was higher in P+ patients compared with controls (5.8 mJ [IQR 4.6–8.9] vs. 4.1 mJ [IQR 2.7–5.4], *P* = 0.0009), despite no significant difference in LVOT pressure gradients on echocardiography between the groups (*Figure 5A*, *Table 2*). Specifically, patients with septal and reverse septal hypertrophy had higher peak systolic KE relative to controls (*P* = 0.0001 and *P* = 0.001) (*Figure 5A*, Supplementary data online, *Table S1*). The high KE was primarily seen in the LVOT, as observed from visual assessment (*Figure 2*). Late diastolic filling, or the A-wave KE, was higher in the P+ patients (2.6 mJ [IQR 1.9; 4.0]) compared with P− cases (1.4 mJ [IQR 1.1–2.1], *P* < 0.0001) and controls (1.9 mJ [IQR 1.4–2.3], *P* = 0.002) (*Table 2*).

Peak systolic KE did not differ between the P− patients and the controls. However, the P− patients had higher early diastolic filling KE compared with the P+ patients (6.3 mJ [IQR 4.4–7.6] vs. 4.1 mJ [IQR 2.8–5.7], *P* = 0.006). The P− patients were also significantly younger than the P+ patients (35 ± 14 years vs. 51 ± 13 years, *P* < 0.0001).

All comparisons of KE remained statistically significant after correcting for multiple testing with a threshold of *P* < 0.0045.

### Systolic and diastolic hemodynamic forces

Systolic longitudinal (apex–base) and transverse HDF were not significantly different between the P+ patients and the controls (*Table 2*). However, the systolic HDF transverse/longitudinal ratio, a marker of inefficient forces, was higher in the P+ patients compared with the P− patients (1.0 [IQR. 0.71–0.95] vs. 0.82, [IQR 0.83–1.1] *P* = 0.01) and the controls (IQR 0.83 [0.72–0.96], *P* = 0.03) (*Figure 5B*, *Table 2*). Subgroup analysis showed a higher systolic HDF ratio in patients with apical hypertrophy compared with the controls (see Supplementary data online, *Table S1*). Systolic longitudinal (apex–base), transverse HDF, and HDF ratio did not differ between the P− individuals and the controls.

Diastolic HDF and HDF ratio did not differ between the P+ patients vs. the P− patients or the controls (*Table 2* and Supplementary data online, *Table S1*). However, the P− patients had lower diastolic HDF (*P* = 0.04) in the lateral wall–septum direction (*P* = 0.04) and lower diastolic HDF ratio compared with the controls (*P* = 0.03).

Group comparisons with diastolic HDF ratio were not statistically significant after correcting for multiple testing.

### Factors that associate with systolic KE and systolic HDF ratio

Among the total cohort of patients with non-obstructive HCM, peak systolic KE correlated with maximum wall thickness (*r* = 0.45, *P* < 0.0001), stroke volume (*r* = 0.55, *P* < 0.0001), stroke work (*r* = 0.60 *P* < 0.0001), and LVOT maximum pressure gradient (*r* = 0.30, *P* = 0.02), but not with EF (*r* = 0.12, *P* = 0.32) or ESC SCD risk score (*r* = 0.13, *P* = 0.38). Systolic HDF ratio correlated with maximum wall thickness (*r* = 0.26, *P* = 0.02), but not with ESC SCD risk (*r* = 0.25, *P* = 0.10), LVOT maximum pressure gradient (*r* = −0.13, *P* = 0.32), stroke volume (SV) (*r* = −0.06, *P* = 0.55), or EF (*r* = −0.03, *P* = 0.81). Only wall thickness, stroke volume, and stroke work remained correlated with systolic KE after correcting for multiple testing with a significance threshold at *P* < 0.0045. Notably, these associations persisted after adjusting for age, sex, and BMI in a multivariate model.

Sarcomeric variant status in the group of all patients was not associated with peak systolic KE or HDF ratio (*P* > 0.10).

### Factors associated with late diastolic KE

Among the patients with non-obstructive HCM, late diastolic (A-wave) KE correlated with maximum wall thickness (*r* = 0.35, *P* = 0.005), stroke work (*r* = 0.38, *P* = 0.0009), and LVOT maximum pressure gradient (*r* = 0.44, *P* = 0.001), but not with EF (*r* = −0.07, *P* = 0.61), SV (*r* = 0.14, *P* = 0.28), ESC risk score (*r* = 0.26, *P* = 0.10), e′ (*r* = −0.12, *P* = 0.37), or E/e′ (*r* = 0.01, *P* = 0.91). Maximum wall thickness, stroke work, and maximum pressure gradient remained correlated with late diastolic KE after correcting for multiple testing using a *P* value threshold of <0.006. These associations persisted after adjusting for age, sex and BMI in a multivariate model.

Sarcomeric variant status in the group of all patients was not associated with late diastolic KE or diastolic HDF or HDF ratio (*P* > 0.10) after adjusting for age.

## Discussion

This study demonstrated that patients with non-obstructive hypertrophic cardiomyopathy (P+) have an increased systolic KE, HDF ratio, and late diastolic KE, along with an increased stroke work relative to pre-hypertrophic sarcomeric variant carriers (P−) and controls. Our findings are intriguing, as transthoracic echocardiography did not reveal significant differences in systolic or diastolic flow measures or in LV ejection fraction (LVEF) among the three groups. Specifically, both the geometry of the ventricle (septal and reverse septal for KE, and apical thickening for systolic HDF ratio) and the extent of hypertrophy in patients with non-obstructive HCM were associated with abnormal systolic KE and HDF ratio, two markers found to predict adverse cardiac remodelling and functional capacity in other cardiac diseases.^12,29^

### Systolic KE, HDF and stroke work

We recently demonstrated that patients with non-obstructive HCM have a unique pattern of ventricular blood flow, where an increase in direct flow proportion (i.e. the proportion of blood that transits the left ventricle within one cycle) is accompanied by a reduction in stroke volume. This contrasted with a healthy pattern of blood flow, where there is close coupling of direct flow proportion and stroke volume.^8^ Notably, this was particularly prominent in individuals with hypertrophy, but the precise mechanisms were unclear. In the present study, we examined KE, HDF and stroke work in a larger cohort to shed further insight into the source of flow inefficiencies in non-obstructive HCM. We found that despite comparable outflow tract velocities on echocardiography between patients and controls, patients with non-obstructive HCM exhibited increased systolic KE, HDF ratio and stroke work relative to the controls. Specifically, systolic KE and HDF were linked to the extent of hypertrophy and accompanied by greater stroke work among the patients. Given that stroke volume, ejection fraction and cardiac output were similar in patients and controls, the increased stroke work was mainly driven by increased afterload in patients, and implies inefficient myocardial work, where more energy is needed to deliver the required amount of blood to the body.

Of interest, systolic KE and HDF ratio were significantly higher in patients with specific cardiac geometries, or patterns of hypertrophy. For instance, patients with reverse septal hypertrophy and septal hypertrophy had higher systolic KE than controls, whereas patients with apical hypertrophy had higher HDF ratio relative to controls, suggesting that altered outflow tract geometry may cause acceleration of outflowing blood to occur more proximally within the LV. While LV KE has been studied in other models of hypertrophy, this is the first comprehensive description of KE, HDF, HDF ratio, and stroke work in HCM. In a study by Peng *et al*.,^30^ systolic KE was also found to be increased in patients with hypertensive heart disease and preserved LVEF, and Steding-Ehrenborg *et al*.^14^ noted that athletes with physiological hypertrophy had normal systolic KE similar to a non-athletic control group. Together, our findings suggest that non-obstructive HCM have distinct patterns of systolic KE and HDF ratio, which have previously been shown to be linked to symptomatic disease states^8,9,11,18,31,32^ and which may allow us to distinguish physiological from pathological hypertrophy in individuals.

### Diastolic KE and HDF

Although all participants in this study had comparable diastolic flow velocities on echocardiography (including E/A ratio, *Table 1*), the P+ patients had increased late diastolic KE (atrial contraction) on 4D-flow CMR when compared with the P− individuals and the controls. These findings highlight the increased sensitivity of KE assessment on 4D-flow CMR to diastolic flow abnormalities. In contrast, we found diastolic HDF to be less sensitive to group differences. For instance, the P+ patients had comparable diastolic longitudinal and transverse HDF when compared with the controls in this study.

Athletes with increased LV mass and volumes have previously been noted to exhibit increased early diastolic peak KE, likely due to an enhanced diastolic function by an increased elastic recoil of the myocardium.^14^ Arvidsson *et al*. recently also noted that early diastolic KE was not determined by LV mass in patients with heart failure with varying ejection fraction,^15^ and the findings in our study are in keeping with this as our results show that early diastolic KE among the P+ patients was no different from that seen in the controls. The precise mechanism for this is unknown, although it is plausible that the stiffer and hypercontractile ventricle in HCM is unable to relax as promptly or effectively as in athletes, and is consequently unable to compensate for the smaller cavity size. Whether group differences in early diastolic KE will become significant following exercise remains to be evaluated.

### Effect of genotype in the absence of hypertrophy

Since the hypercontractile phenotype in HCM is in part mediated by energy-costly changes in sarcomeric myofilament proteins,^33^ we evaluated if increased systolic KE, HDF ratio, or diastolic parameters are seen in the P− patients (i.e. carriers of the pathogenic sarcomeric variants without LV hypertrophy). The P− patients were noted to have higher early diastolic KE and lower diastolic transverse HDF and HDF ratio than the P+ patients, although the P− patients were 16 years younger than the P+ patients. When adjusting for differences in age, there was no significant association between diastolic KE or HDF or HDF ratio and sarcomeric variant status. This is consistent with previous studies that have also noted a possible age-dependence for diastolic KE in normal aging and in the setting of non-hypertrophic LV dysfunction,^15,17^ which may explain the difference in diastolic KE, HDF, and HDF ratio across the groups.

### Clinical utility

Emerging pharmacological therapies for HCM are primarily targeted at alleviating outflow tract pressure gradients on echocardiography in patients with symptomatic obstructive HCM,^1^ while patients with non-obstructive HCM have limited therapeutic options. Reliable haemodynamic measures comparable to outflow tract velocities are currently lacking in the latter group, making it challenging to precisely monitor the effects of disease-specific therapies. Our data suggest that 4D-flow CMR HDF ratio and KE may provide more sensitive measures of hemodynamic perturbations than standard echocardiography, enabling better monitoring of the effects of such therapies on LV haemodynamics and stroke work in non-obstructive HCM. Intraventricular hemodynamics measured with 4D-flow CMR were informative of systolic and diastolic flow inefficiencies without the need for provocation manoeuvres, and have the potential to accelerate diagnosis and timely referral for novel disease-specific therapies, such as myosin ATP-ase inhibitors. In the present study, there was a weak, but non-significant, positive correlation between systolic HDF ratio, late diastolic KE, and ESC risk. This was not surprising in view of the inclusion of individuals with normal LVOT gradients with low ESC risk. Further studies are therefore needed to examine the clinical and prognostic significance of abnormal HDF ratio or KE in symptomatic HCM patients.

### Limitations

Patients were categorized as non-obstructive based on LVOT pressure gradient assessments at rest and following Valsalva manoeuvre. Exercise provocation was not employed in our study, and thus exercise-inducible increased LVOT pressure gradients cannot be excluded in our cohort. The majority of patients in this study were asymptomatic (83% in NYHA class I), and further studies are needed to evaluate the clinical significance of KE and HDF in symptomatic patients. Patients with HCM were analysed in relation to a control cohort matched on the group level to all patients; however, the subgroup of P− individuals were younger than the P+ patients and the controls, and had a larger proportion of women than the P+ patients. Although the proportion of patients with sarcomeric positive HCM in this study is higher than in other published HCM studies, this is to be expected since enrolment was restricted to non-obstructive HCM cases.

While 4D-flow CMR data are seemingly limited by low spatial and temporal resolution, previous studies have validated the accuracy and reproducibility using phantom and *in vivo* measurements for intraventricular and great vessel 4D-flow measurements.^27,34^ Clinical implementation of 4D flow has historically been challenging due to relatively long acquisition times, but modern acceleration techniques have reduced the acquisition time to 4–6 min with preserved data quality^27,35^ and 4D-flow CMR remains the gold standard for analysis of intracardiac KE and HDF.

## Conclusion

Despite comparable flow velocities on echocardiography, patients with non-obstructive HCM exhibited higher stroke work, higher systolic KE and HDF ratio, and late diastolic KE relative to controls. 4D-flow CMR provides sensitive measures of hemodynamic inefficiencies in non-obstructive HCM, with potential for application in therapeutic clinical trials and clinical surveillance.

## Supplementary Material

qyae074_Supplementary_Data

## Data Availability

The data underlying this article will be shared on reasonable request to the corresponding author.

## Lead author biography

This research project is a collaboration between the University of Oxford and Lund University. Karin Pola is currently a PhD student at Lund University and a physician in Clinical Physiology at Skåne University Hospital Lund, and she has worked as a visiting PhD student at the University of Oxford. The overall aim of her research is to gain a greater understanding of cardiac mechanics and hemodynamics, and to find new diagnostic tools and improve the clinical care for patients with cardiac conditions.
