# Supplementary material for: Flow inefficiencies in non-obstructive HCM revealed by kinetic energy and hemodynamic forces on 4D-flow CMR
Source: Eur Heart J Imaging Methods Pract. 2024 Jul 16;2(3):qyae074. doi: 10.1093/ehjimp/qyae074 (PMC11350944; doi:10.1093/ehjimp/qyae074)
Supplement: qyae074_Supplementary_Data [file qyae074_supplementary_data.docx]

**Data supplement**

**Supplemental Table 1. Kinetic energy and hemodynamic forces in hypertrophy phenotype subgroups.** Data in median [interquartile range] or mean and [standard deviation]^#^ and (p value) controls.

|  | **Septal  (n=14)** | **Reverse septal (n=14)** | **Apical  (n=9)** | **Concentric (n=11)** |
| --- | --- | --- | --- | --- |
| Age, years^#^ | 52 [16] (0.4) | 49 [8.2] (0.7) | 54 [13] (0.3) | 49 [16] (0.8) |
| Maximum wall thickness (mm)^#^ | 19 [4.1] | 24 [4.0] | 20 [5.5] | 20 [5.4] |
| Stroke work (J)^#^ | 1.4 [0.14] (*0.01*) | 1.4 [0.36] (0.1) | 1.3 [0.31] (0.4) | 1.2 [0.32] (0.6) |
| **Peak kinetic energy (mJ)** | | | | |
| Systole | 7.1 [5.5;9.6] (*0.0001*) | 7.8 [4.1;10] (*0.001*) | 4.7 [3.6;5.7]  (0.6) | 5.1 [4.1;7.6]  (0.1) |
| Early diastole | 5.5 [3.8;6.7]  (0.2) | 4.2 [2.7;5.5]  (0.8) | 3.3 [2.5;5.5]  (0.4) | 3.4 [2.0;5.4]  (0.2) |
| Late diastole | 3.3 [2.1;4.6]  (*0.006*) | 3.4 [2.1;4.5] (*0.003*) | 2.2 [1.7;3.2]  (0.4) | 2.7 [1.9;3.5] (0.07) |
| **Peak kinetic energy indexed to end-diastolic volume (mJ/l)** | | | | |
| Systole | 39 [32;48] (*0.0001*) | 41 [27;57] (*0.005*) | 29 [25;30] (0.3) | 31 [25;40] (0.1) |
| Early diastole | 30 [23;33] (0.6) | 24 [14;27] (0.07) | 21 [15;33] (0.5) | 16 [14;29] (0.06) |
| Late diastole | 19 [12;23] (*0.04*) | 16 [11;23] (*0.04*) | 12 [9.8;20] (0.3) | 18 [9.5;21] (0.1) |
| **Hemodynamic forces (N)** | | | | |
| **Longitudinal (apex-base)** | | | | |
| Systole | 0.24 [0.17;0.26] (*0.03*) | 0.23 [0.16;0.27] (0.2) | 0.14 [0.12;0.16] (0.09) | 0.18 [0.14;0.23] (0.6) |
| Diastole | 0.11 [0.089;0.14] (0.9) | 0.12 [0.099;0.16] (0.5) | 0.092 [0.070;0.12] (0.08) | 0.12 [0.077;0.15] (0.5) |
| **Transverse (lateral wall-septum)** | | | | |
| Systole | 0.20 [0.15;0.24] (*0.02*) | 0.22 [0.12;0.26] (*0.03*) | 0.14 [0.094;0.18] (0.9) | 0.17 [0.14;0.21] (0.3) |
| Diastole | 0.027  [0.020;0.035]  (0.5) | 0.029 [0.023;0.032] (0.8) | 0.022 [0.016;0.032] (0.3) | 0.027 [0.018;0.040] (1.0) |
| **Transverse (inferior-anterior)** | | | | |
| Systole | 0.036  [0.026;0.060]  (0.1) | 0.041 [0.025;0.050] (0.07) | 0.027 [0.015;0.042] (0.9) | 0.029 [0.022;0.044] (0.6) |
| Diastole | 0.025  [0.022;0.030]  (0.5) | 0.031 [0.025;0.040]  (0.2) | 0.026 [0.023;0.033] (0.3) | 0.022 [0.016;0.026] (0.6) |
| **Hemodynamic force ratio (transverse/longitudinal)** | | | | |
| Systole | 0.88 [0.74;1.2] (0.4) | 1.0 [0.81;1.2] (0.05) | 1.1 [0.98;1.2] (*0.002*) | 0.92 [0.74;1.1] (0.5) |
| Diastole | 0.33 [0.24;0.45] (0.5) | 0.34 [0.27;0.42] (0.5) | 0.45 [0.30;0.52] (0.4) | 0.36 [0.29;0.42] (0.5) |
| *Mean and peak early and late diastolic HDF could not be computed reliably, and therefore root mean square pan-diastolic HDF and HDF ratio were used. | | | | |

**Supplemental Figure 1. Method agreement measurements of kinetic energy and hemodynamic forces** on 4D flow cardiovascular magnetic resonance using standard versus papillary-border contouring of the left ventricular cavity. Data is presented for 5 HCM and 2 controls in systole and diastole as peak kinetic energy and root mean square hemodynamic forces. Measurement differences are calculated as standard contouring subtracted by papillary-border contouring. Filled horizontal lines are bias, and dashed lines are 95% limits of agreement.
